# A position statement on mental health in the post-2015 development agenda

**DOI:** 10.1186/1752-4458-8-28

**Published:** 2014-07-07

**Authors:** Julian Eaton, Ritsuko Kakuma, Alexandra Wright, Harry Minas

**Affiliations:** 1 CBM International, West Africa Regional Office, Lomé, Togo; 2 Global and Cultural Mental Health Unit, Centre for Mental Health, Melbourne School of Population and Global Health, The University of Melbourne, Parkville, Australia

**Keywords:** Global mental health, Millennium development goals, Low and middle income countries, Development

## Abstract

**Background:**

The Millennium Development Goals have guided development co-operation in the 15 years up to 2015, achieving some significant progress in the priorities on which they focused. As the framework for the post-2015 development agenda is negotiated, this article reviews the evidence for the place of mental health in broader development issues that have already been outlined in the run-up to 2015.

**Discussion:**

If mental health is going to be recognised as having an essential role in development, there needs to be a consensus on priorities for advocacy. Various key issues emerged from a survey of stakeholders in the Movement for Global Mental Health (MGMH), leading to a Position Statement, which is now available for use by advocates. The priorities that emerged were increasing access to mental health services, and addressing human rights abuse, stigma, and exclusion.

**Summary:**

Mental health is a cross-cutting issue, and including it in frameworks for action will increase the likelihood of achieving global priorities for development such as poverty reduction, economic development, improved health, and ensuring the most vulnerable in society are not left behind.

## Introduction

The Millennium Declaration [1], adopted by the UN General Assembly in September 2000, provided the framework for the Millennium Development Goals (MDGs) which gave a common direction to global development cooperation for the fifteen years to 2015. They were able to garner a strong consensus from a wide range of development actors, including governments in developed and less developed nations, non-governmental organisations, donor agencies and associated organisations. The result was a significant improvement in international cooperation that had a measurable impact on development of poorer countries and fragile states [2].

As we approach the end of the MDGs programme it is possible to assess its impact. In his foreword to the 2013 MDGs report the UN Secretary-General, Ban Ki-Moon, observes that “Significant and substantial progress has been made in meeting many of the targets-including halving the number of people living in extreme poverty and the proportion of people without sustainable access to improved sources of drinking water. The proportion of urban slum dwellers declined significantly. Remarkable gains have been made in the fight against malaria and tuberculosis. There have been visible improvements in all health areas as well as primary education” [2]. However, global progress has been uneven, and there still remains a lot to be achieved, particularly in sub-Saharan Africa.

In preparation for the end of the MDGs programme a great deal of work has been done on framing the post-2015 development agenda. The themes and priorities for the next phase of development effort have arisen out of two main parallel processes. The UN Conference on Sustainable Development, or ‘Rio +20’, in June 2012 laid out a set of priorities which aimed to reduce poverty, advance social equity and ensure environmental protection. These Sustainable Development Goals (SDGs) have a strong environmental focus, and emphasise global economic justice, including the price that will have to be paid to avert significant climate change [3]. In parallel to this, the United Nations embarked on a major consultation exercise at various levels to develop a framework that will take over from the MDGs [4]. A landmark event was the publication of the report of the High-Level Panel of Eminent Persons on the Post-2015 Development Agenda [5] which outlines a framework for the main themes of the agenda. Thematic papers have also been prepared on health, education, and other areas by relevant UN agencies. A process of public consultation at country level was co-ordinated by the UN Development Programme (UNDP), and many organisations and associations advocating in particular areas developed position papers highlighting their views on priorities that should be considered in the debate.

One relevant example of this is the global disability movement, which was able to present a common position statement through a well organised, co-ordinated effort by international disability associations [6]. This emphasised the importance of the framework being inclusive in all areas, and they have achieved some success on this aim. In contrast, despite the consultation process nearing its final stages, there had been no co-ordinated and concerted effort to ensure that mental health issues were incorporated into the development agenda in a way that stakeholders in the field felt was appropriate or useful for reducing the global burden of mental disorders.

## Emerging themes

Following intensive debate and lobbying, various themes have emerged that are likely to shape the framework that will result from the process described.

The High Level Panel of Eminent Persons pointed out that the original MDGs enabled considerable achievements, and that there should be a continuation of the Millennium Declaration’s three principles of human rights, equal opportunities and sustainability. They also pointed out a number of areas that require stronger emphasis in the post-2015 agenda:

1. The lack of focus on vulnerable persons and most marginalised resulted in their relative neglect. The Panel therefore suggested adopting the central principle of ‘leave no-one behind’. This theme is reinforced in the determination that equity should be a major parameter to monitor success. Whereas in the first MDGs targets usually looked at aggregate change the Panel pointed out that there must be a measure of equity in future to avoid the neglect of vulnerable groups.

2. The Panel was clear that it saw economic development as the driver for reducing poverty, but that this must be sustainable. Damage to ecosystems and climate change present huge hurdles to development, especially in the poorest countries where a high proportion of the population is closely dependent on the land. Economic development that further damages ecosystems is therefore counter-productive [7].

3. The initial MDGs were sometimes perceived as setting up a false dichotomy between donor and recipient countries. The framework in the future is likely to emphasise shared global challenges that require co-ordinated effort to address them. There is already a shift away from development aid as the major driver of change, towards improved governance that allows the benefits of national resources to be more fairly distributed within nations. Three quarters of the world’s poor live in middle income countries, and greater peace and security would significantly reduce the suffering, ill health and poverty resulting from violence and displacement.

4. The environmental and social determinants of poverty and ill health need to be addressed. Violence, war and insecurity often have their roots in poverty and injustice, and are major impediments to progress. Corruption, illegal outflows of capital, poor management of extractive industries, and unfair international trade agreements deprive the poorest countries of the opportunity to use their own resources to drive their own development.

Clearly, this final point is particularly relevant to the field of mental health where social determinants play a central and persistent role as risk factors for mental ill health, and at the same time mental ill health makes it harder for people to rise out of poverty and reduce vulnerability to exploitation, abuse and violence [8]. Strategies to counteract mechanisms that reinforce poverty and inequalities is one way of starting to address these otherwise persistent barriers to development.

## The health agenda

Priorities in health will be influenced by demographic changes, disease burden shifts, and lifestyle changes [8]. These transitions are likely to result in non-communicable diseases (NCDs) taking centre stage in the post-2015 agenda as they are predicted to become an even greater cause of mortality and disability in the future. While this does not mean that NCDs will ‘replace’ other disease priorities, it is likely that environmental and behavioural determinants of health will be better recognised. Health will be seen more as a means to achieving broader objectives of development than being expressed in a series of narrow single-issue targets. Health is likely to be included in terms of targets that take a more holistic life-course approach, with evidence-based, system-wide interventions that recognise environmental influences and see health as a human right [9].

Health needs to be positioned, therefore, as an essential contributor to sustainable development, for example by focusing on economic development and poverty reduction through the improved productivity of a population in good health. Health can also be recognised as an outcome affected by progress in economic, development and social sectors. Health is also immediately relevant to people’s lives and seen as a powerful lever for mobilising support on issues that might otherwise seem, to the public and politicians, as being less tangible, e.g. climate change.

Mental health has a strong bearing on many of the themes above. Of particular relevance is the growing importance of happiness and wellbeing [10]. This is likely to be a significant theme within the post-2015 agenda, having been positioned as an essential measure of human progress in the last few years. In the influential 2013 World Happiness Report mental ill health is identified as the single biggest determinant of happiness and life satisfaction, contributing much more than physical health, age, gender, or income and employment [10]. Wellbeing has significant political traction at present, and given that mental health is recognised as playing a major role, and has a strong evidence-base for cost-effective interventions, this presents an important opportunity for advocacy for inclusion in the post-2015 agenda.

## Mental health in the development agenda

It is unlikely that mental health will be prioritised as an identifiable vertical goal. However, its relevance in the NCD agenda has been explicitly recognised in the UN General Assembly Declaration on the Prevention and Control of Non-communicable Diseases [11]. This is not only because, by definition, mental disorders are NCDs, but because of the strong reciprocal relationship between mental ill health and other NCDs. NCDs (e.g. diabetes and cardiovascular disease) increase the risk of developing mental disorders, especially depression, and mental ill health is a risk factor for development of several NCDs. Importantly, untreated mental ill health has a negative impact on NCD treatment outcomes [12]. Mental health is therefore not only a sub-goal in the NCD field, but should be fully integrated into the service structures for NCDs, which otherwise are likely to have their impact limited by untreated mental ill health.

The lack of a specific ‘mental health goal’ in the original MDGs, or even mention of mental health as a relevant issue, was often considered as an obstacle to prioritisation of mental health in development work in the initial decade of the 21st century. However, mental health is relevant in many of the MDGs, for example poverty, human rights, and the physical health goals [13]. In many ways this actually provides many avenues for inclusion of mental health in development programmes across a range of sectors, a strategy that can guide advocacy.

It is increasingly recognised that health is an essential component of overall sustainable development. Support for health programmes is no less important for development than is support for programmes in education, improved governance, water and sanitation, economic development and a host of other development priorities. It is no less clear that mental health is essential for positive sustainable human development, and that improved population mental wellbeing is an outcome of such development. Consideration of mental health also advances the objectives of equity, universal coverage, wellbeing, a holistic and life-course approach to health and human rights.

## The MGMH position statement: mental health is essential to achieve sustainable development

To quote the World Health Organization (WHO) discussion paper on positioning health in the post-2015 development agenda - “multiple consultative and deliberative processes are already taking place. Given limited time, scarce resources and a fast-moving target, a strategic approach to influencing outcomes is needed” [8]. This is also the case for mental health.

Since the process of development of the MDGs, global mental health has emerged as a more clearly distinct field, and has made progress in strengthening an evidence-base and understanding better the main issues that must be addressed [14]. Some progress has also been made in raising the profile of the field as a development issue requiring action, defining messages and priorities, and to a lesser extent, organising stakeholders to promote these messages. The Movement for Global Mental Health (MGMH) [15] was established after the publication of the Lancet series on Global Mental Health in 2007. It is a loose membership body, mainly working through a website to provide a communication platform for members. As a multi-stakeholder forum with individual and institutional members from academia, civil society, professional disciplines, and service users and carers, it is ideally positioned to advocate for a place for mental health in the post-2015 agenda. In order to focus the message and facilitate coherent advocacy it was decided to develop a Position Statement on mental health in the post-2015 development agenda.

The working draft of an MGMH position statement on mental health in the post-2015 agenda, developed by members of the MGMH Advisory Group, incorporated many of the themes and priorities discussed in this paper. The draft was posted on the MGMH website [15] and circulated via the membership email list, with an invitation for input from members. Comments and suggestions, received from 54 individual members and associations, were incorporated into a second draft. Suggested revisions included: emphasising the positive role mental health plays in supporting other development initiatives; the cross-sectoral relevance of mental health; highlighting inclusion, service user empowerment and human rights; recognising the impact of child abuse and gender violence; and specifically aligning the aims and implementation strategies with the NCD community, the WHO Mental Health Action Plan, and the UN Convention on the Rights of Persons with Disabilities.

This draft was then presented at the 3rd MGMH Summit in Bangkok, Thailand, on August 22nd, 2013 [16]. Input from members at the Summit was then incorporated into a final version (Additional file 1) circulated for use by members on the website and through email.

The foundation for a common position must represent the views of as many relevant stakeholders as possible. The World Federation for Mental Health (WFMH) and MGMH collaborated in a survey of 500 civil society organisations, which resulted in ‘The People’s Charter for Mental Health’ [17]. In the process of developing the MGMH Position Statement the responses from stakeholders concerning main priorities were surprisingly similar to those received during the development of the People’s Charter, suggesting that the community of actors in global mental health have successfully united behind common themes for action. It is important to note, however, that those who respond to surveys are generally those who are already committed to the basic principles of the organisation seeking their opinion, and dissenting views may not have been captured. On the other hand, both WFMH and MGMH are deliberately broad-based organisations, and aim to include service users among their respective memberships.

The emergence of common themes in advocacy for mental health as part of the wider development agenda is reflected in shared priorities for action and a growing consensus about implementation. The Comprehensive Mental Health Action Plan 2013–2020 [18], developed by the WHO following the relatively weak emphasis on mental health in the UN Special Session on NCDs in 2011 [11], has been widely welcomed. Outcome indicators from the action plan and the availability of instruments such as the WHO Assessment Instrument for Mental Health Services (WHO-AIMS) and published reports from more than 100 countries and territories [19] will provide a basis for development of mental health indicators and targets for the post-2015 development goals.

As we have seen above, an approach that is broader than a focus just on health will be necessary within the post-2015 framework. Such a holistic approach is likely to be welcomed by parts of the service user community who have criticised the emphasis of WHO initiatives on scaling up services and the relative lack of prioritisation of human rights issues and inclusion. The Convention on the Rights of People with Disabilities has provided important new avenues for campaigning and practical action in this area, as has the new WHO QualityRights Programme [20].

The Statement does not include everything that needs to be done to advance the mental health of populations, but it does identify core components and practical priorities for action. It articulates with other major programmes and initiatives. It is, we think, an effective instrument for advocacy. But an instrument, if it is to have an impact, must be effectively used. We therefore urge all who would wish to see mental health fully incorporated into post-2015 development programmes to use the statement in their advocacy for such an outcome. The message that mental health is essential as a contributor to and as an outcome of sustainable development, and the principle that no-one must be left behind, must at every opportunity be conveyed to decision-makers with conviction and clarity.

## Competing interests

The authors declare that they have no competing interests.

## Authors’ contributions

All authors contributed to development of the Position Statement discussed. They jointly conceived the article. JE prepared the first draft. All authors contributed to subsequent drafts and all have seen and approved the final version.

## Supplementary Material

Additional file 1 MGMH Position Statement on the post-2015 MDGs. Click here for file
