# Supplementary material for: A position statement on mental health in the post-2015 development agenda
Source: Int J Ment Health Syst. 2014 Jul 7;8:28. doi: 10.1186/1752-4458-8-28 (PMC4104399; doi:10.1186/1752-4458-8-28)
Supplement: Additional file 1 — MGMH Position Statement on the post-2015 MDGs. [file 1752-4458-8-28-S1.pdf]

Mental Health is Essential to Achieve Sustainable Development

This position statement presents the current best evidence in the field of global mental health and highlights the positive contribution that attention to mental health can make to the global development agenda. It was prepared with the participation of individual and organisational members of the Movement for Global Mental Health.

We call upon the United Nations and other parties who are developing the Post 2015 Framework and implementation plan, and those who will implement proposed activities, to include the following three specific elements, all of which are essential components of the global development agenda.

1. ***Promote protection of human rights and prevent discrimination against people with mental illness and psychosocial disability***
   - Promote implementation of the UN Convention on the Rights of Persons with Disabilities, strengthen human rights protections and prevent discrimination.
   - Enable people with mental illness and psychosocial disability to make decisions about their own welfare, and to participate fully in processes affecting them.
   - Ensure equal access for people with mental illness and psychosocial disability to health, education, livelihood and other development projects, to ensure that people who are among the most vulnerable are not left behind.

2. ***Bridge the massive mental health treatment gap and improve access to health and social care***
   - Support the full implementation of the WHO Comprehensive Mental Health Action Plan.
   - Integrate mental health treatment and psychosocial support services into Non-Communicable Disease services.
   - Ensure parity with physical illnesses in access to treatment and care.
   - Ensure full access for people with mental illness and psychosocial disability to universal health coverage.

3. ***Explicitly integrate attention to mental health into development initiatives***
   - Ensure sustained attention to the most vulnerable groups, including people with mental illness and psychosocial disability, in development activities in the fields of health, education, economic development and human rights.
   - Integrate measures of mental wellbeing in monitoring and evaluation of development effectiveness.

In order to ensure that best practice in mental health is fully integrated into the post-2015 developmental goals, specific targets related to mental health and wellbeing must be set. The practical suggestions in this Position Statement will support efforts of all parties to achieve the overall aims of the framework. The community of stakeholders in global mental health is ready to work with development partners to support such efforts.

Global mental health facts

- **Good mental health** is central to overall health and well-being, as reflected in the WHO definition of health. It is an essential component of community and national development
- **Happiness and life satisfaction.** Recent research, highlighted in the 2013 World Happiness Report, shows that mental ill health is the leading determinant of happiness and life satisfaction, contributing more than physical health, age, gender, income or employment

- **Premature and avoidable mortality**. Life expectancy for those with serious mental illness is 10-15 years less than for those with good mental health. The impact of mental illness on life expectancy is comparable to or greater than that of more widely appreciated contributors to premature mortality such as smoking, diabetes and obesity. Suicide is now recognised as a leading cause of death among young people in many countries
- **Vulnerability and exclusion**. People with mental illness and associated psychosocial disability are among the most vulnerable members of society, particularly in low and middle-income countries. People with mental disorder continue to experience systematic exclusion from social and economic life.
- **Disability**. Mental disorders account for 13% of the total global burden of disease, and 37% of Years Lived with Disability.
- **Human rights violations** and discrimination are a common experience among people with mental illness and psychosocial disability. Examples include containment (e.g. chaining, imprisonment without charge), exclusion from social and family life and educational and employment opportunities, institutional denial of property and inheritance rights and the right to political participation, and being subjected to medical and other treatments and interventions without appropriate consent.
- **Insufficient investment**. The 1-2% of health budgets spent on mental health care by governments in low income countries is neither proportionate to the burden, nor sufficient to provide full access to evidence-based treatments.
- **Treatment gap**. Globally the great majority of people with mental illness do not have access to affordable and effective treatment and social support services. The treatment gap is greatest in low and middle-income countries, where up to 90% of those with mental illness receive no treatment or care. This is an affront to basic human rights and a lost opportunity make a cost-effective investment in human health and development.
- **Mental illnesses are treatable.** Treatments for mental illnesses that occur throughout the life-course are effective, shortening illness episodes, reducing relapse and recurrence, and reducing associated disability. Effective and culturally appropriate services that meet local needs are a good investment.
- **Integrated care.** There is increasing evidence for how effective, affordable, comprehensive mental health services can be integrated into general health systems and community settings. Such interventions have been tested in low and middle income countries, where they are effective, even when delivered by non-specialists.
- **Intervening to prevent poverty.** Mental health interventions are pro-poor, and can break the cycle of mental ill health and poverty that affects households and families, results in missed life opportunities, social exclusion, underemployment, and high healthcare costs.

WHO Comprehensive Mental Health Action Plan

The Comprehensive Mental Health Action Plan was adopted by the WHO World Health Assembly in May 2013. This followed recognition of mental health as an essential component of the global health agenda, including within Non-Communicable Diseases. The vision of the action plan is a world in which mental health is valued, promoted and protected, mental ill health is prevented and affected persons are able to exercise the full range of human rights and to access high quality, culturally-appropriate health and social care in a timely way to promote recovery, all in order to attain the highest possible level of health and participate fully in society and at work free from stigma and discrimination.

The Comprehensive Mental Health Action Plan will reinforce growing efforts by members of the Movement for Global Mental Health and others to improve access to health and social care, challenge abuse of human rights, and to promote social inclusion for people with psychosocial disabilities in all parts of the world.

There can be no effective development without mental health

The High-Level Panel of Eminent Persons on the Post-2015 Development Agenda, in its report published on 30 May 2013, determined that "…we must go beyond the MDGs. They did not focus enough on reaching the very poorest and most excluded people." In light of this, we would highlight the particular relevance of mental health to the identified transformative shifts in the development agenda.

Addressing mental health is a human rights issue and the overall goal of sustainable development can only be achieved with proper consideration for the mental health dimension. The social and economic costs of failure to attend to mental health will render activities aimed at achieving the post-2015 development agenda less effective.

The High-Level Panel concluded that the post-2015 agenda "needs to be driven by five big, transformative shifts". Mental health is highly pertinent to all of these:

Transformative shifts	Mental health
**1. Leave no one behind**	Focus on the *very poorest and most vulnerable groups*<br>• Even within global co-operation for development, people with mental illness and psychosocial disability have often been left behind. Mental health services and psychosocial care must be mainstreamed into development programmes, which will be stronger if there is meaningful participation of those they affect in their design, implementation and evaluation
**2. Put sustainable development at the core**	*Sustainable and inclusive economic growth*<br>• There is strong evidence to show that poverty and mental ill health form a vicious cycle, and that mental health and psychosocial support interventions can break this cycle, leading to improved economic welfare in individual, household, community and national life
**3. Transform economies for jobs and inclusive growth**	Ensure that all people can *contribute to the economy*<br>• People with mental disorder and psychosocial disability, who are most commonly at the most productive age of economic life, face *barriers and discrimination to employment*, even though many are able to contribute skills to economic growth. There is need to ensure equal opportunities for a dignified livelihood.
**4. Build peace and effective, open and accountable institutions for all**	The effect of *conflict and violence*:<br>• With respect to national and international public policy, there is clear evidence that poverty, income inequality, economic shocks, conflict and complex emergencies, and high per capita alcohol consumption are all important contextual factors that erode mental capital. Conversely, peace, economic, physical and social security, and social justice are conditions that allow mental capacity to flourish. A long term perspective is needed in order to contribute to individual and communal well-being and the promotion of peace and stability
**5. Forge a new global**	Inclusive *global partnerships*<br>• More global funds for development need to be devoted to mental health and psychosocial support, reflecting the

**partnership**	contribution of mental illnesses to the global burden of disease and the negative impact on individual and communal wellbeing. An approach to improve mental health and psychosocial wellbeing needs to be integrated as a cross-cutting issue in the health sector (including child and maternal health care, HIV, and NCDs) and in community development strategies. Psychosocial disability should be fully integrated into the global and national agenda for the rights of persons with disabilities, including the UN Convention on the Rights of Persons with Disabilities (CRPD)

Specific interventions to achieve these transformative shifts

- **Education**: Half of all adult mental ill health starts before the age of 16. Early recognition and intervention significantly reduces subsequent, potentially lifelong, disability. Consideration of mental wellbeing in education systems, including educating children about protecting their own health, should be part of education programmes. Gender-based violence and child abuse have profound psychological consequences and should be addressed as a major cause of mental ill health. Targeted approaches to reduce domestic and gender-based violence and child abuse are essential to reduce the long-term effects on mental health.

- **Healthcare**: A *huge treatment gap* currently exists. Up to 90% of people with mental ill health worldwide do not receive any treatment or care. Cost-effective models for provision of high quality mental health care have been shown to be effective, and are able to bridge this gap, giving people with mental health care needs choices that do not currently exist for most. Care must be taken to ensure that such interventions meet local needs and are culturally appropriate. Families and carers who currently carry a significant burden should be supported

- **Non Communicable Diseases (NCDs)** will increasingly contribute to the burden of disease in the future. Mental and substance use disorders constitute the single largest burden of disease group among the NCDs, and fit extremely well into systems for health promotion, as well as treatment models developed for NCDs. They are similar in often being persistent, requiring long-term treatment in decentralised locations such district and primary health care units. Mental health conditions often co-exist with other NCDs such as diabetes, heart disease and cancer. The effective treatment of mental ill health can substantially reduce the severity and health burden due to the other NCDs, and improve long-term outcomes. We propose to work closely with those in the field of NCDs so as to provide access to comprehensive and integrated services that have been shown to support better recovery.

- **A data revolution for sustainable development**. In the last 10 years efforts to strengthen the integrity of global data systems for mental health (particularly for health systems and human rights) have resulted in a greater understanding of availability and gaps in resources, and the reality of lives of people with mental health needs. Specific indicators and targets for mental health are needed in the post-2015 framework (for example within NCDs) to ensure that mental illness is not neglected in future as it has been in the past.

The Movement for Global Mental Health (MGMH) is a network of about 3,000 individual and institutional members from more than 60 countries, which brings together service users, carers, policymakers, academics, professionals, service providers, civil society organizations and disabled peoples' organizations, from low- and middle- and high-income countries, to promote mental health as an integral part of overall global development
